# Special issue on “Self‐management challenges of chronic pain experiences in children and young people: A developmental perspective”

**DOI:** 10.1002/pne2.12087

**Published:** 2022-09-02

**Authors:** Line Caes, Abbie Jordan

**Affiliations:** ^1^ Division of Psychology, Faculty of Natural Sciences University of Stirling Stirling UK; ^2^ Department of Psychology University of Bath Bath UK; ^3^ Centre for Pain Research University of Bath Bath UK

A dominant focus in the treatment of chronic pain in children and young people is placed on self‐management. Self‐management can be broadly defined as “an individual's ability to manage the symptoms, treatment, physical and psychological consequences, and lifestyle changes inherent in living with a chronic illness” (p. 178).^1^ Evidence has highlighted the importance of self‐management in terms of improving an individual's physical and psychosocial functioning.^1^, ^2^

Developing adequate self‐management skills is of particular importance in later childhood and adolescence (further referred to as young people). This is because, at this timepoint, individuals begin taking greater responsibility for self‐management of their chronic pain while parents typically reduce their involvement in managing young people's pain.^3^ Critically, supporting the development of effective self‐management at this point in development and into emerging adulthood is important since effective self‐management of pain can offset potential lifelong pain‐related disability.^4^

Many evidence‐based treatment programmes for chronic pain promote the necessary skills to self‐manage pain episodes such as using relaxation, acceptance‐based approaches, problem‐solving, continued and consistent engagement with exercises, and medication adherence.^5^ However, there is no standardized operationalization of self‐management in the context of pediatric chronic pain and how self‐management (and its impact on young people) should be assessed. Furthermore, there is a limited understanding of how the developmental challenges associated with the onset of adolescence and young adulthood (e.g., development of a sense of identity, increased focus on peer relationships) impact the experience of pediatric chronic pain treatments, including self‐management, in young people and their parents. Such a comprehensive understanding of self‐management experiences from a developmental perspective is crucial to overcoming the challenges faced by healthcare professionals to engage young people with self‐management.^6^, ^7^

This special issue collected four cutting‐edge articles which expand upon the existing knowledge concerning the impact of developmental challenges that young people and their parents may face with regard to engaging with effective self‐management strategies for managing their chronic pain. Articles within the special issue adopt a range of methodologies (i.e., mixed methods design, qualitative design, intervention development, and narrative review) across various key stages of young people's development, including the stage of emerging adulthood (18–30 years^4^). It is noteworthy to mention that across the articles, a different operationalization for young people was adopted, ranging from the typical narrow adolescent focus (11–17 years adopted by Ghio et al.^8^) to wider operationalizations (e.g., 8–18 years^9^ or 12–22 years of age^10^). A lack of consensus regarding the ages associated with adolescence and young adulthood is consistent with the developmental literature. Notably, the adoption of a wider operationalization is in accordance with an argument made to extend the ages of adolescent onset and completion to acknowledge later completion of developmental milestones such as leaving the family home.^11^ Furthermore, acknowledging the broader social context of development and pediatric chronic pain, all bar one of the articles includes a focus on the role of parents in young people's self‐management of chronic pain.

The special issue commences with an innovative qualitative exploration of coping goals in young people with Juvenile Idiopathic Arthritis and their parents,^8^ focusing on how young people's coping goals match onto their cognitive and emotional perspectives of chronic pain. The findings illustrate how self‐management support for young people needs to consider the centrality of preserving young people's social identity as their goal, through either maintaining a “normal” lifestyle or managing their pain. Interestingly, based on the data, the authors developed a coping framework, illustrating how young people and parental cognitive and emotional perspectives on pain map onto these two different coping goals. This framework not only expands our understanding of young people's coping goals in managing their chronic pain but is also critical in driving forward future research systematically exploring coping experiences in young people with chronic pain from a motivational perspective and provide important targets for self‐management interventions.

The developmental challenges in coping with chronic pain, and the centrality of the social context were further illustrated in the article by Jones et al.^10^ In this article, Jones and colleagues used several forms of data (diaries and multiple interviews) to innovatively adopt a longitudinal approach to examine adolescent and parental temporal perceptions of developmental challenges in the context of young people living with chronic pain. The authors of this study identified fluctuations in the role that pain played in both restricting and providing opportunities to young people, especially with respect to engaging with their social environment and perceptions of autonomy. Not only do these findings highlight two important targets for self‐management interventions (i.e., social development and autonomy), but the findings stress how self‐management support needs to be attuned to/be flexible in response to naturally occurring changing perceptions and maturity across development.

The article by Smith and Logan^9^ addresses the commonly observed problem of engaging adolescents within intensive interdisciplinary pain treatment (IIPT) programmes for chronic pain (which include self‐management support). Smith and Logan^9^ comprehensively describe the development of a pediatric telehealth intervention to improve young people's and their parent's readiness and engagement with such IIPT programmes: Promoting Readiness and Engagement in Pain Rehabilitation (PREPaRE). The PREPaRe intervention follows the evidence‐based principles of motivational interviewing and was developed through consultation with experts, and further refined through a pilot study with young people with chronic pain and their parents. The final version of the PREPaRE telehealth intervention comprises four modules, addressing the need for change, building commitment, identifying barriers, and strengthening commitment. While further evaluation is warranted, especially with respect to how engaging with PREPaRE impacts engagement with and impact of IPPT programmes, the pilot study revealed high levels of engagement and treatment satisfaction with PREPaRe. The study findings highlight the importance of targeting interventions specifically around preparing individuals to effectively engage with interventions which treat chronic pain in young people.

Lastly, the special issue ends with a narrative review^4^ focusing on an often‐overlooked sample in the context of pediatric chronic pain: emerging adults (aged 18–30 years of age). In particular, and in line with the centrality of the social context highlighted in the majority of articles within this special issue, the review reflects upon the role of parents in the context of managing pediatric pain. In particular, the article focuses on helicopter parenting, a term which describes parental over‐involvement in their child's life. This narrative review offers important insights into the societal shifts made with respect to helicopter parenting and its impact on young people with chronic pain yet also provides key clinical recommendations on how to address helicopter parenting styles when providing self‐management to emerging adults.

In sum, these four papers raise important issues concerning developmental challenges that young people and parents face when considering and engaging with self‐management of pediatric chronic pain. Critically, these papers highlight the need of adopting a developmental approach to self‐management in pediatric pain and appreciating how challenges (e.g., development of autonomy, changing social context, and reduced parental responsibility for self‐management) may fluctuate over time. Much as the often quoted ‘children are not little adults’ statement highlights the distinctness of children from adults, it is critical to appreciate the unique and detailed nature of changes in development across adolescence and early adulthood when understanding and promoting effective self‐management processes in young people who live with pain.
